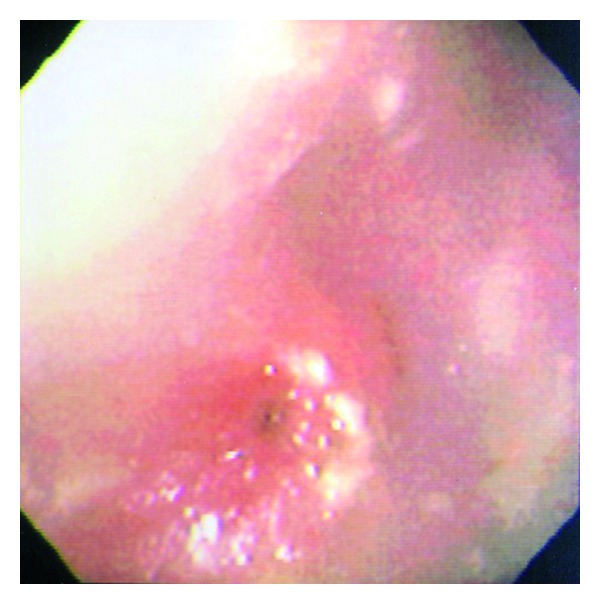# Two Cases of *Helicobacter pylori*-Negative Gastric Outlet Obstruction in Children

**DOI:** 10.1155/2011/749850

**Published:** 2011-08-25

**Authors:** Raza A. Patel, Susan S. Baker, Wael N. Sayej, Robert D. Baker

**Affiliations:** ^1^Division of Pediatric Gastroenterology, Hepatology, and Nutrition, University of Utah and Primary Children's Medical Center, Salt Lake City, UT 84113, USA; ^2^Digestive Diseases & Nutrition Center, Women & Children's Hospital of Buffalo, Buffalo, NY 14222, USA; ^3^Division of Digestive Diseases, Hepatology & Nutrition, Connecticut Children's Medical Center, Hartford, CT 06106, USA

## Abstract

Gastric outlet obstruction (GOO) in children is most commonly caused by idiopathic hypertrophic pyloric stenosis. Prior to proton pump inhibitors and H2 blockers, peptic ulcer disease (PUD) secondary to *H. pylori* was a cause of GOO. Both patients presented with a history of weight loss, vomiting, and abdominal pain. Their diagnosis of PUD and GOO was made by EGD and UGI. *H. pylori* testing was negative for both on multiple occasions but still received *H. pylori* eradication therapy. Patient 1 after failing pharmaceutical management underwent surgery for definitive treatment. Patient 2 underwent six therapeutic pyloric dilations before undergoing surgery as definitive treatment. These cases suggest that GOO secondary to PUD occurs in the absence of *H. pylori* infection and surgical management can provide definitive therapy.

## 1. Introduction

Gastric outlet obstruction (GOO) is a well-identified entity leading to the clinical symptoms of abdominal pain and vomiting. Prior to the introduction of proton pump inhibitors (PPIs) and H2 blockers (H2Bs) peptic ulcer disease (PUD) secondary to *H. pylori* infection was recognized as a common cause of GOO. In infants, GOO is usually caused by idiopathic hypertrophic pyloric stenosis (IHPS) [1], although other anatomic abnormalities can cause obstruction; in children, GOO is less common [2].

## 2. Case 1

Patient 1 is an 11-year-old male who presented with a 3-year history of postprandial vomiting and abdominal pain, 1.5 kg weight loss, and plateau in height. Weight at presentation was 28.3 kg (7th percentile), height 144.5 cm (54th percentile), and BMI 13.5 (<5 percentile). On examination only asthenia was noted. The complete blood count, electrolytes, erythrocyte sedimentation rate, stool elastase, and 72-hour fecal fat were normal. Stool examinations for *H. pylori *antigen, hemoccult, bacteria, and viruses were negative. 

An esophagogastroduodenoscopy (EGD) with a standard endoscope showed retained food and liquid in the stomach despite 18 hours of fasting. Visual examination showed thickened mucosal folds along the lesser curvature and stenosis of the pylorus with no visible ulcers. The patient was started on lansoprazole 30 mg daily. 

Rapid urease test (*Campylobacter-like organism, *CLO test, Kimberly-Clark, Roswell, GA) of the gastric antrum and fundus was negative, upper gastrointestinal series (UGI) showed a narrowed pyloric channel, and computed tomography (CT) of the abdomen/pelvis and pyloric ultrasound were normal. The patient became asymptomatic, and the lansoprazole was stopped after 6 months of therapy.

**Figure 1:**
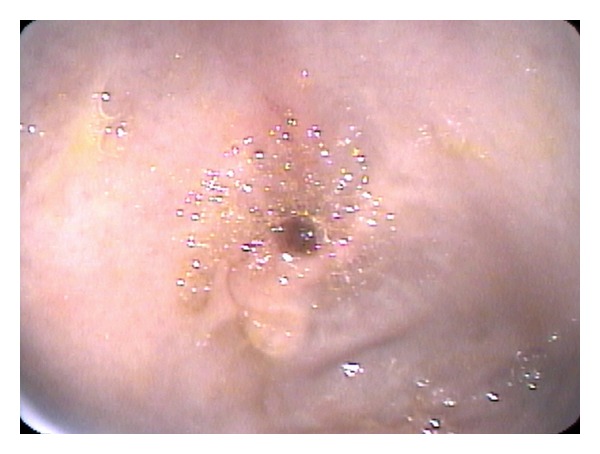


**Figure 2:**
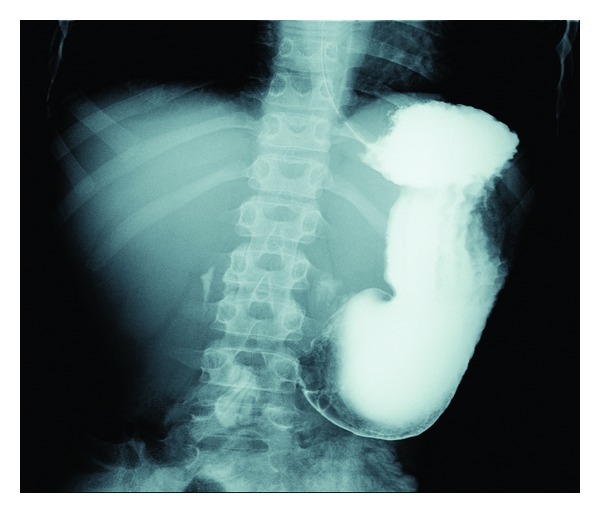


Seven months later, the patients symptoms recurred, and lansoprazole, 30 mg/day, and nutritional supplementation were started. One month later, the patient lost 2.5 kg. Repeat EGD showed stenosis of the pyloric sphincter; lumen diameter estimated at 3 mm (Figure 1), gastrin was 27 pg/mL (normal 0–100), and a repeat CLO was negative. Pathology from the pyloric channel showed necrotic tissue with neutrophilic and fibrinous exudates; adjacent gastric mucosa showed active inflammation. The patient was admitted for high-dose proton-pump inhibitor treatment (pantoprazole 40 mg IV bid) and parenteral nutrition. UGI showed a narrow pyloric channel with ulceration and cicatrization (Figure 2). Barium remained in the stomach after 18 hours. 

An EGD eight months after initial presentation showed a small pylorus that could not be traversed with a 6 mm endoscope (Pentax EG1870K, Pentax Medical, Montvale, NJ). Histology of the duodenum showed benign mucosa containing a lymphoid follicle and no active inflammation. The antrum showed mild active gastritis. Serum studies and colonoscopy were not suggestive of Crohn's disease. The patient was referred for surgical intervention. In the operating room a dilated stomach and a 1–1.5 cm thickened area of the pyloric channel were observed. A Jaboulay pyloroplasty was performed. The resected section showed an ulcer, with normal smooth muscle and hypertrophic nerves. The surface epithelium was replaced by granulation tissue. The lamina propria showed mild active chronic inflammation. No etiologic source for the hypertrophy was identified.

Three months following surgery, 10 months after initial presentation, the patient was asymptomatic; his weight was 41.2 kg (48th percentile) and height 148 cm (38th percentile).

## 3. Case 2

Patient 2 is a 15-year-old female who presented with a 2-month history of vomiting and weight loss. Laboratory evaluation prior to referral included normal *H. pylori* IgG and abdominal ultrasound. A large pyloric channel ulcer was seen on UGI. Weight was 53.8 kg (50% tile), height 160 cm (35% tile), and physical exam unremarkable. 

She was treated for *H. pylori*. The EGD demonstrated a narrowed pyloric channel (Figure 3) and mucosal abnormalities. The pyloric channel was dilated with CRE Wire-guided 6-7-8 180 cm pyloric balloon dilator (Boston Scientific, Natick, MA). Biopsies showed normal histology of the esophagus and antrum and no tissue from the duodenal bulb. Repeat ultrasound revealed GOO caused by thickening of the pylorus channel length 3.2 cm, thickness 1.2 cm. Biopsies were negative for *H. pylori, *and serum gastrin and thyroid levels were normal. Over the next 6 weeks the patient had a total of 7 diltations, each resulting in transient improvement in symptoms. Surgical referral was made following the second dilatation, and following a failed seventh dilatation she underwent a Billroth I gastrectomy with preservation of the vagal nerve. Pathology of the stenosed area showed muscular hypertrophy with no identifiable etiology. Following surgery all symptoms of GOO resolved, and follow-up EGD 18 months after surgery showed no ulcer and no scaring.

## 4. Discussion

Excluding infancy GOO is a rare condition. The rate of GOO in the pediatric/adolescent population is unknown. Etiologies of GOO after the neonatal period include peptic ulcer disease; gastric tumors, including adenocarcinoma, lymphoma [3], and gastrointestinal stromal tumors; infections, such as tuberculosis; eosinophilic gastroenteritis [4] and infiltrative diseases, such as amyloidosis [5]. Most of the information on the successful treatment of GOO comes from the adult literature. The approach to management in adults has been applied to children without evidence to show its effectiveness in children. 

*H. pylori* positive GOO is successfully treated with PPI and antibiotic therapy [6–8]. *H. pylori* negative peptic ulcer disease (PUD) is usually managed with dilatation and PPI. Lau et al. [9] in a case series of 41 patients with benign GOO reported that 6 of 10 patients who had a negative CLO test responded to NG suction and IV PPI therapy, and three patients developed recurrent obstruction and required surgery. In a series of 23 patients with PUD-related GOO Cherian [10] identified 3 patients with idiopathic obstruction and all three improved on acid suppression therapy alone. 

In addition to PPI therapy benign GOO can be treated with serial dilations [11, 12] however [9, 13–15] patients requiring repeat dilatations eventually require surgery as definitive therapy. The role of *H. pylori* therapy in PUD-related GOO is less clear although eradication is warranted as a primary intervention [16]. Lam et al. [17] reported that in *H. pylori*-negative patients undergoing dilatation, the rate of recurrent obstruction was 4/11 and was not significantly different from the rate of recurrent obstruction in the *H. pylori*-positive group. Gibson et al. [13] concluded that patients with *H. pylori*-negative PUD leading to GOO should have early surgery because of high recurrence of obstruction. 

In children, cases of GOO outside of infancy have been reported that are non-PUD-related [18]. Edwards et al. [19] identified 29 cases of gastric outlet obstruction during a 23-year period. Nineteen of these patients had duodenal ulcers, and all underwent a surgical procedure. The presence of *H. pylori* was not routinely assessed, and these patients were not treated with PPI and/or dilitations. Yen and Kong [20] identified 11 children without IHPS. Six patients had PUD-related obstruction, and 3 were *H. pylori* negative. In the *H. pylori* negative cohort only one patient required surgical intervention; the other two patients had resolution with PPI therapy only. 

We conclude that GOO secondary to ulceration can occur in the absence of *H. pylori* infection. Our patients demonstrate that medical management was palliative but definitive management was surgical.

## Figures and Tables

**Figure 3:**